# Moving the Needle in KMT2A Rearranged Pediatric B-Cell Acute Lymphoblastic Leukemia: Newer agents and novel approaches

**DOI:** 10.46989/001c.141198

**Published:** 2025-06-27

**Authors:** Anwesha Ray, Aditi Jain, Mona Vijayaran, Steve Thomas, Jayastu Senapati, Mukul Aggarwal

**Affiliations:** 1 Lady Hardinge Medical College, New Delhi, India; 2 Dept. of Haematology, Safdarjung Hospital, Vardhaman Mahavir Medical College, New Delhi, India; 3 Dept. of Clinical Haematology, Sanjay Gandhi Postgraduate Institute of Medical Science, Lucknow, India; 4 Dept. of Haematology, Sri Ramachandra Medical College, Chennai, India; 5 Dept. of Leukemia, The University of Texas MD Anderson Cancer Center, Houston, TX, USA; 6 Dept. of Haematology, All India Institute of Medical Sciences, New Delhi, India

**Keywords:** KMT2A, blinatumomab, revumenib, CAR T-cell therapy, Pediatric B-ALL

## Abstract

Pediatric B-cell acute lymphoblastic leukemia (B-ALL) has been the poster child of progressive success in the development of leukemia therapy. Among the genomically defined high-risk subtypes of B-ALL are those with *KMT2A*-rearrangement (r) which are associated with inferior outcomes with chemotherapy-based approaches. *KMT2A*-r ALL is most common in the infantile period but can be seen beyond it and has remained a therapeutic challenge. Recent clinical trials have shown a significant improvement in response rates and survival outcomes in infantile and pediatric non-infant patients with *KMT2A*-r B-ALL when treated with blinatumomab-containing regimens. A single course of sequential blinatumomab added to Interfant-06 chemotherapy led to an exceptional improvement in 2-year disease free survival to 82% compared to 49% from historical chemotherapy only approach. In the salvage settings the use of tisagenlecleucel chimeric antigen receptor (CAR) T-cell therapy has led to high response rates and durable remissions in pediatric *KMT2A*-r B-ALL. Recently, inotuzumab ozogamicin was approved in pediatric (>1 year) relapsed/refractory B-ALL, widening immunotherapy-based salvage options. However, the efficacy of inotuzumab in *KMT2A*-r B-ALL remains questionable, given lower CD22 expression in this ALL genotype. Additionally, the approval of menin inhibitors like revumenib in *KMT2A*-r pediatric acute leukemias provides another treatment option in the salvage setting for this high-risk pediatric B-ALL subtype. These targeted agents are positively altering the treatment approaches and outcomes in pediatric *KMT2A*-r B-ALL, and the use of better residual disease monitoring with next generation sequencing might further help to refine treatment approaches in such high-risk pediatric ALL.

## Introduction

Acute lymphoblastic leukemias (ALL) show a diverse range of genetic abnormalities that determine the ontogeny of the leukemia, course of the disease, and response to treatment. One such genomic aberration involves the rearrangement of lysine (K)-specific methyltransferase 2 A *(KMT2A)* gene, located on chromosome 11q23.^1^ With more than 100 translocation partners, *KMT2A* rearrangement (*KMT2A-r*) can lead to ALL, acute myeloid leukemia (AML), and mixed phenotypic acute leukemia (MPAL), with dismal prognosis across all categories.^2–5^ While 75% of infantile ALL and 10-15% of pediatric AML demonstrate *KMT2A*-r, it is rare in non-infant childhood ALL (around 5-10%).^6^ The responses of such leukemias to standard chemotherapy are poor, with lower rates of measurable residual disease (MRD) negativity and higher rates of relapse in both the pediatric and adult population.^2–5^ Most of the *KMT2A-r* ALL present as B-ALL but can rarely be T- ALL.^7^

*KMT2A-r* ALL can often have co-expression of myeloid and lymphoid markers which poses diagnostic challenges.^8–10^ Expert pathology review is warranted to discern the correct diagnosis. Response and MRD rates are further dependent on the partner gene involved in the translocation with *KMT2A*; the Ponte di Legno Childhood ALL Group study of *KMT2A*-r pediatric ALL, showed MRD negativity rate of 51% in (4;11)(q21;q23)/*KMT2A::AFF1* rearrangement compared to 75% in t(10;11)(p12;q23)/ *KMT2A::MLLT10*.^5^ The five-year event-free survival (EFS) rate was 69% for the full cohort of *KMT2A*-r ALL, while it was 65% for *KMT2A::AFF1* B-ALL.

*KMT2A* encodes the protein MLL1, which, when bound to menin (a product of tumor suppressor gene MEN1), upregulates the HOX genes, which causes leukemogenesis.^8,11,12^ Menin inhibitors that target *KMT2A-r* acute leukemias are currently being studied, and revumenib was recently approved for relapsed/refractory (R/R) acute leukemias with *KMT2A* translocations in both adult and pediatric populations for AML, ALL, and MPAL.^13^

**Figure 1.**
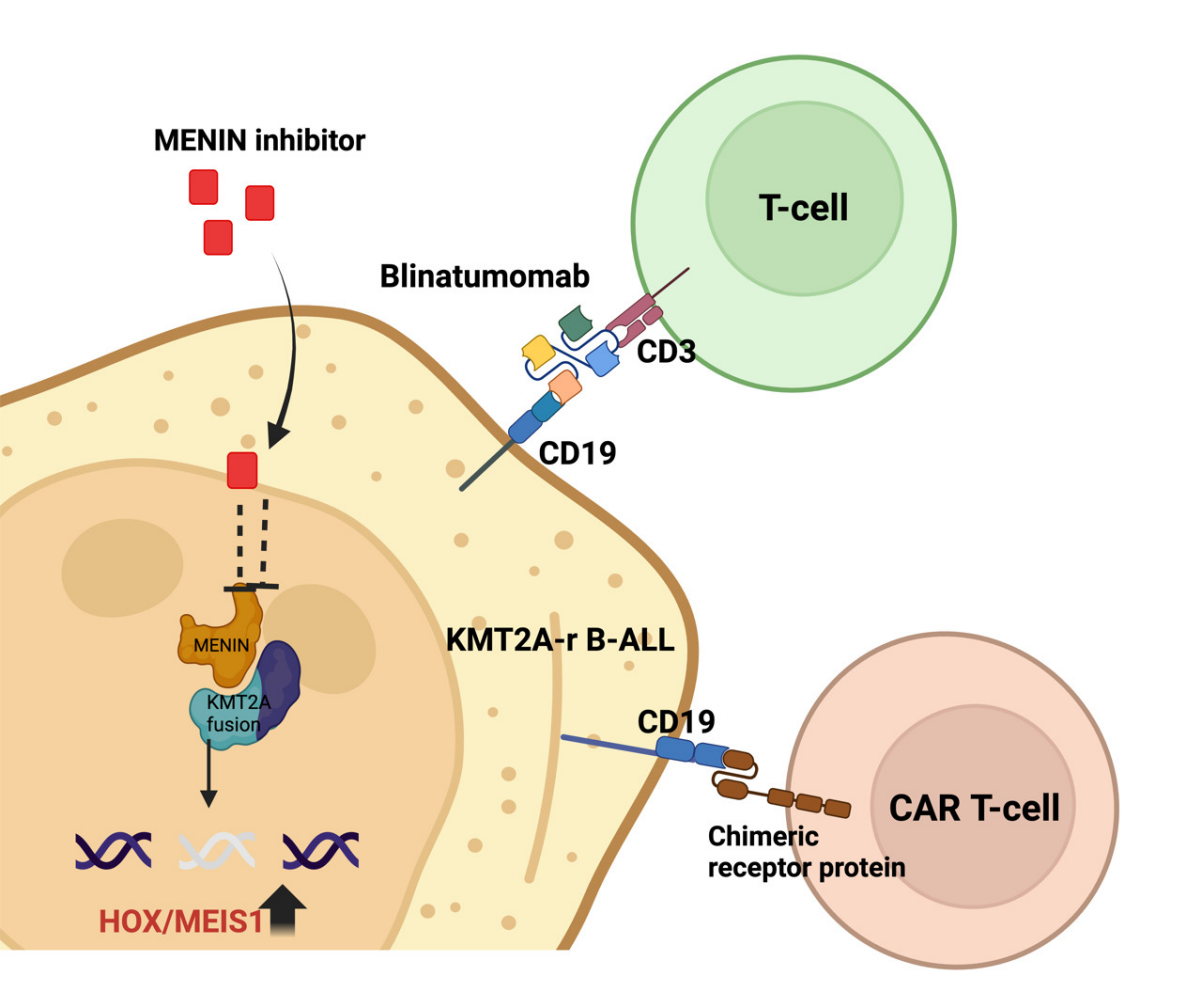
Site of action of targeted therapies in *KMT2A*-r B-ALL Figure made on Biorender.com

Targeted therapy using the CD19-directed bispecific T-cell engager (BiTE) blinatumomab has led to drastic improvements in *KMT2A*-r B-ALL.^14,15^ The use of such targeted agents in pediatric *KMT2A*-r B-ALL marks a significant step towards the management of such high-risk leukemias **(Figure 1).** Furthermore, CD-19 directed chimeric antigen receptor T-cell (CAR T) therapy has been extensively evaluated in R/R pediatric B-ALL and tisagenlecleucel is an approved agent in this setting. Inotuzumab ozogamicin (InO) was also approved about a year ago in pediatric (age >1 year) R/R pediatric B-ALL and widens the treatment armamentarium in the salvage settings for high-risk pediatric B-ALL, though it might have limited efficacy in *KMT2A*-r B-ALL. In this review we describe the improving outcomes of *KMT2A*-r pediatric B-ALL with these novel targeted agents and cellular therapy approaches.

**Table 1.** Studies with targeted therapies in pediatric *KMT2A*-r B-ALL

**Author**	**Study arm**	**Study cohort**	**Chemo backbone**	**Response**	**Survival outcomes**
**Blinatumomab**					
Gupta et al^16^	Randomized Chemo vs Chemo+ Blina (two 28-days cycles) as consolidation	Newly diagnosed Standard risk B-ALL with average or high risk of relapse N= 1440, Median age= = 4.3 years	Augmented BFM	Not relevant	MFU: 2.5 years, 3-year DFS= 96% (with blina) vs 88% (with chemo alone) 3-year OS= 98.4% (with Blina) vs 97.1% (Chemo alone)
		*KMT2A*-r B-ALL, n=25 (15 in blina and 10 in chemo arm)			3-year DFS=85% vs. 74%
Sluis et al^15^	Single arm Chemo+ Blina (single course) as post-induction therapy	Newly diagnosed *KMT2A*-r B-ALL Infants < 1year age N=30	Interfant-06	MRD negative CR=100%	MFU: 26.3 months, 2-year DFS= 82% 2-year OS= 93%
**CAR T-cell therapy**					
Leahy et al^17^	Retrospective, pooled analysis of 5 different CAR T-cell products	R/R B-ALL with high-risk genomics (n=231) Age 1-29 years *KMT2A*-r, n=25 (infants, n=13)	Not applicable	CR=92%	All *KMT2A*-r 2-year RFS=46% 2-year OS= 60% *KMT2A*-r Infants 2-year RFS=67% 2-year OS= 62%
Ghorashian et al^18^	Retrospective, pooled analysis from Europe with Tisa-cel	R/R B-ALL, N=38 Infused and response evaluable, n=28 *KMT2A*-r, n=29	Not applicable	CR/CRh=24 (86%)	1-year EFS=41% 1-year OS= 84%
Colleen et al^19^	Retrospective analysis from Pediatric Leukemia Adoptive Therapy trials 2 separate CAR T-cell products	R/R *KMT2A*-r infantile B-ALL, N=19 Cells manufactured in 18 Infused in 17	Not applicable	MRD negative CR/CRi= 16 (94%)	1-year PFS= 75% 1-year OS= 77% 3-year LFS, censored for HSCT= 64%
Moskop et al^20^	Retrospective analysis from Pediatric Real World CAR Consortium with Tisa-cel	Infants with R/R B-ALL (*KMT2A*-r, n=12)	Not applicable	MRD negative CR/CRi= 9 (64%)	6-months EFS=48% 6-month OS=71%

**Abbreviations:** Blina, blinatumomab; B-ALL, B-cell acute lymphoblastic leukemia; r, rearrangement; MFU, median follow up; DFS, disease free survival; OS, overall survival; r, rearranged; MRD, measurable residual disease; CR, complete remission; EFS, event free survival; LFS, leukemia free survival; HSCT, hematopoietic stem cell transplantation

## Blinatumomab in pediatric B-ALL

Blinatumomab received accelerated United States Food and Drug Administration (US FDA) approval in 2014 based on results from the open label phase-2 clinical trial by Topp et al, which evaluated the drug in adult patients with R/R CD19-positive (+) Philadelphia (Ph)- negative B-ALL.^21^ The drug led to complete remission (CR)/CR with incomplete hematological recovery (CRh) rates of 43%, and was overall safe. The TOWER trial was a confirmatory phase-3 randomized control trial (RCT) which evaluated blinatumomab in adult patients with R/R Ph-negative B-ALL and showed a significant improvement in survival compared to standard of care (SOC) chemotherapy (7.7 months versus 4.0 months).^22^ This, along with the phase-2 ALCANTRA trial, which evaluated blinatumomab in adults with R/R Ph-positive B-ALL and showed CR/CRh rates of 36%, led to the full approval of blinatumomab by FDA and expansion of label for treatment of adult patients with R/R Ph-positive B-ALL.^23^ Based on the results of the BLAST trial, blinatumomab was also approved for MRD eradication in children and adults with MRD positive B-ALL.^24^

The first important study with blinatumomab in the pediatric setting was the open label phase 1/2 MT103-205 clinical trial which included patients <18 years of age with Ph-positive or negative CD19+ B-ALL.^25^ Blinatumomab led to CR rates of 39% in patients treated at the phase-2 dosing along with reasonable safety.^25^ Notably in this study, blinatumomab showed potent efficacy in R/R *KMT2A*-r B-ALL: 5 of 8 patients aged <2 years and with *KMT2A*-r achieved a CR. This study formed the basis for the inclusion of pediatric patients in the 2014 FDA accelerated approval of blinatumomab, along with adults. Blinatumomab showed significant efficacy as salvage therapy in several studies of pediatric patients with R/R B-ALL.^26–28^ Additionally, blinatumomab has been found as a safe and efficacious alternative in patients intolerant to chemotherapy, having persistent MRD or refractory post chemotherapy-based induction/consolidation.^29^

In June 2024, blinatumomab was approved as consolidation in both pediatric (≥ 1 month of age) and adult B-ALL, based on the ECOG 1910 trial.^30,31^ This trial studied 2 cycles of blinatumomab as consolidation therapy in adult patients with MRD-negative status post-induction, compared to SOC consolidation chemotherapy. At about 43 months of follow up, blinatumomab showed superior 3-year OS (primary endpoint) of 85% compared to 68% in the SOC arm. Although the outcomes of pediatric standard risk B-ALL are good with chemotherapy and lead to high cure rates, a recent study showed further improvement when blinatumomab was added to consolidation therapy.^16^ In a phase 3 RCT by Bhatia et al, pediatric patients with standard risk B-ALL (white blood cell [WBC] <50x 10^9^/L and age 1-10 years) but with average or high-risk of relapse (using a composite of baseline genomics and post-induction MRD) were randomized to receive chemotherapy alone or 2 non-subsequential courses of intravenous blinatumomab. Among 1440 patients randomized, the 3-year disease-free survival (DFS) was 96% with blinatumomab compared to 88% with chemotherapy-based regimen.^16^ Traditional blinatumomab toxicities like cytokine release syndrome (CRS) were not commonly observed.

## Blinatumomab in Infantile and pediatric *KMT2A*-r ALL

Infantile ALL is a rare but challenging disease with poor outcomes; long-term OS rates are around 50-60%.^32,33^ Age <6 months is a particularly high-risk factor in *KMT2A*-r infantile ALL and, traditionally, these infants were poor prednisolone responders, with outcomes being further poor in those with presentation WBC counts >300 x 10^9^/L. In the Interfant-99 study, patients with all these risk factors had 4-year EFS of 20% compared to 45% for others.^32^ Moreover, in the Interfant-99 study, infantile ALL did not show any significant improvement in outcomes with allogeneic hematopoietic stem cell transplantation (HSCT), except in the subgroups with poor prognostic features mentioned above. Almost 75% of infantile ALL has a *KMT2A*-r with inferior outcomes: 5-year EFS < 40% compared to *KMT2A*-germline that has 5- year EFS >75%.^2,32,34^ *KMT2A*-germline pediatric ALLs are usually less commonly associated with high WBC and central nervous system (CNS) leukemia at presentation.^35^ Though infantile *KMT2A-r* ALL can have both lymphoid and myeloid markers, the addition of myeloid-directed consolidation therapy to standard ALL chemotherapy has not led to a significant improvement in outcomes, except for those with poor response to induction therapy.^2,36^ Most relapses (90%) occur within 2 years of treatment and 66% within the first year.^2^ After multiple attempts over decades, a recent development has been the successful addition of blinatumomab in the treatment of infantile *KMT2A-r* ALL.^37^ Blinatumomab was added as a single course, post-induction (chemotherapy induction as per Interfant-06 protocol) at a dose of 15mcg/m^2^ body surface area per day as a 28-day continuous infusion.^37^ All patients achieved an MRD-negative remission. The 2-year EFS rate with blinatumomab showed a drastic improvement from 49.4% (as seen in the historical Interfant-06 trial) to 81.6%, and the 2-year OS rate increased from 65.8 % to 93.3%.^37^ The addition of blinatumomab was tolerable with no major therapy-related toxicity.^37^ Blinatumomab is now being studied in infants with *KMT2A-r* ALL in the ongoing Interfant-21 trial (NCT05327894).^38,39^

In the AALL1731 trial, 25 patients with *KMT2A*-r B-ALL were included (high-relapse risk subgroup), of whom 15 were treated in the blinatumomab arm.^38^ The 3-year DFS rates were significantly better with blinatumomab, at 85%, compared to 74% in patients treated in the chemotherapy arm.^16^ Notably, blinatumomab is known to cause a myeloid switch of *KMT2A*-r B-ALL to AML.^40^ This might be more relevant when blinatumomab is used as a monotherapy or with minimal chemotherapy. Overall, in multiple prospective studies, blinatumomab containing therapy have shown a significant improvement in outcomes in infantile and pediatric non-infantile *KMT2A*-r B-ALL.

## CAR T-cell therapy in *KMT2A*-r pediatric B-ALL

Chimeric antigen receptor (CAR) T-cell therapy is an efficacious treatment option in R/R CD19+ B-ALL.^41^ Tisagenlecleucel (Tisa-cel) was the first and remains the only approved CAR T-cell therapy approved in pediatric R/R B-ALL (up to 25 years of age) based on the results from the ELIANA trial.^42^ In the long-term 39-month follow-up of 79 patients treated on this trial, 3-year EFS and OS rates of 44% and 63% were reported, while 3-year relapse free survival (RFS) censored for subsequent ALL therapy was 52%.^43^ Though this trial included patients with *KMT2A*-r B-ALL, their outcomes were not separately reported.

In a retrospective analysis, pediatric and young adults (1-29 years) with CD19+ B-ALL were treated in the salvage settings with 5 separate CAR T-cell products (investigational CAR T and tisa-cel). Among 25 patients with *KMT2A*-r B-ALL, the 2-year RFS and OS rates were 46% and 60%, respectively, while the same rates in 13 infants with *KMT2A*-r B-ALL were 67% and 62%, respectively.^17^ In a multicenter European study evaluating 38 patients (<3 years of age) with R/R B-ALL planned for tisa-cel, 35 got the infusion and 29 had a *KMT2A*-r B-ALL; the 1-year EFS and OS rates were 69% and 84%, respectively.^18^ Rates of severe grade CRS were seen in 14% of patients, with no severe grade neurotoxicity. In another study including only infantile *KMT2A*-r B-ALL, Colleen et al reported on the feasibility and efficacy of CAR T-cell therapy in 19 infants treated on the Pediatric Leukemia Adoptive Therapy (PLAT) trials.^19^ Eight patients (41%) were in first relapse and the rest in second or greater relapse; 8 (41%) had been treated previously with blinatumomab and 9 (47%) had been previously transplanted. Two separate CAR T-cell products were used. CAR T-cell was successfully manufactured in 18/19 (95%) patients and 17 (89%) got the infusion. Sixteen of 17 (94%) treated patients achieved CR/CR with incomplete count recovery (CRi), all of whom became MRD negative. The 1-year progression free survival was 75% and the OS 77%. The products were well tolerated with only 1 grade 3 CRS (14/17, 82% all grade CRS) and low rates of immune effector cell associated neurotoxicity (ICANS) (2/17, 12%, all ≤ grade 2). Among these 17 patients, 14 were alive at approximately 2 months post CAR T-cell infusion, 7 (50%) of whom received a consolidative HSCT. However, even with censoring for HSCT, the 3-year leukemia free survival (LFS) for all 16 responding patients was a promising 64%.

In another study including 14 infants with R/R B-ALL (12 *KMT2A*-r) from the Pediatrics Real-World CAR consortium, 5 of whom were in first relapse and the remaining in primary refractory or ≥ second relapse, 9 (64.3%) achieved an MRD-negative CR after tisa-cel infusion (8/12 [75%] with *KMT2A*-r).^20^ The 6-month EFS and OS were 48% and 71%, respectively, and only 1 patient was transplanted post tisa-cel. Interestingly, 4 patients with *KMT2A*-r had a myeloid lineage switch after tisa-cel in this study. CRS was seen in 11 (79%) patients with 3 events (21%) being ≥grade 3. These important studies show that CAR T-cell therapy is an efficacious and safe treatment option in high-risk *KMT2A*-r infantile ALL. However, myeloid lineage switch after CD19 targeted therapy poses a challenge in *KMT2A*-r B-ALL, and needs to be carefully watched out for.

Despite the formidable efficacy of CAR T-cell therapy in high-risk pediatric B-ALL like *KMT2A*-r ALL, access to cellular therapy remains a major deterrent in several geographic regions across the world, due to issues of accessibility, availability and affordability. Additional questions remain on the optimal time point of use of CAR T-cell therapy (as part of consolidation in frontline, MRD eradication or salvage therapy) and whether allogeneic HSCT is needed in patients with *KMT2A*-r ALL after CAR T-cell therapy, especially in those who receive such agents in the salvage setting. Deeper MRD monitoring by next generation sequencing (NGS) based approaches at a level of detection of 10^-6^ can identify patients at high risk of relapse after CAR T-cell therapy and, possibly, the need for allogeneic HSCT to prevent morphologic relapse.^44^

## Inotuzumab ozogamicin *KMT2A*-r pediatric B-ALL

InO is a CD22- targeting antibody drug conjugate that was initially approved for adult patients with R/R B-ALL and subsequently for pediatric patients with R/R B-ALL. In the registrational Children’s Oncology Group phase 2 study (COG AALL1621; NCT02981628), a CR rate of 42% was achieved among 53 treated patients aged 1-22 years, along with an MRD negative CR rate of 96% and median duration of CR of 8.2 months. While this adds to treatment options in the salvage settings for pediatric B-ALL, InO might not be an efficacious agent in *KMT2A*-r B-ALL, given that the lower CD22 expression in these patients leads to poor InO binding and activity.^45,46^ None of the 6 patients with *KMT2A*-r B-ALL in the above study achieved a composite CR (CRc) with InO. Even in adults, *KMT2A*-r B-ALL is associated with relatively lower CD22 expression and inferior outcomes with InO based salvage therapy.^47,48^ However, InO-based combination approaches could be considered in those patients with *KMT2A*-r B-ALL who have adequate CD22 expression (>90%). Concerns of InO-related hepatic sinusoidal obstruction syndrome remain relevant, especially in patients who proceed to allogeneic HSCT after InO. However, these rates have been significantly reduced in adults with a fractionated InO dosing approach, using InO containing combinations rather than monotherapy, and modifying HSCT conditioning regimens.^49^

## Menin inhibitors in *KMT2A*-r ALL

As mentioned earlier, the interaction of menin with *KMT2A* and subsequent overexpression of *HOX* genes is responsible for the pathogenesis of acute leukemia with *KMT2A*-r. Thus, inhibiting menin can potentially stop the uncontrolled proliferation of undifferentiated cells.^8,11^ Revumenib is a menin inhibitor that has recently been approved in R/R acute leukemia with *KMT2A*-r based on the AUGMENT-101 trial.^13^ This phase 1b/2 trial included 94 patients (age 30 days or more) with *KMT2A*-r acute leukemia, in which 78 were AML,14 ALL, and 2 were of ambiguous lineage.^13^ Efficacy analysis was done among 57 patients, which showed a combined rate of CR and CR with partial hematologic recovery (CR+ CRh) of 23%, CRc rate of 44%, and overall response rate (ORR) of 63%. At a median of 6 months of follow-up, the median OS was 8 months in the complete cohort. Among those patients who achieved CRc, 15 out of 22 (68%) became MRD negative.^13^ From the pediatric aspect, 23 patients treated on this study were <18 years of age with a median age of 4 years, and the efficacy evaluable population included 13 patients.^13^ They were heavily pre-treated with a median of 4 prior lines of therapy, which included venetoclax-based therapy in 8 (62%) patients, CAR T-cell in 2 (15%) , and allogeneic HSCT in 6 (64%).^50^ The rate of CR/CRh was the same as that of adults (23%, 3/13) and the median time to CR/CRh was 2.3 months.^50^ The CRc rate was 39% (5/13), and the ORR was 46% (6/13). Among the patients who achieved CRc, 60% (3/5) had no detectable MRD. Some of the common side effects across all age groups (safety evaluable population; n=94) included nausea (44%), differentiation syndrome (28%), and QTc prolongation (26%).^13^ Neither differentiation syndrome nor prolonged QTc interval cause discontinuation of revumenib. Only 12.8% of patients had treatment-emergent adverse events that led to drug discontinuation.

Several other menin inhibitors are in development (ziftomenib, bleximenib, enzomenib etc.), and their efficacy in pediatric MEIS/HOX-dependent leukemias (such as *KMT2A*-r ALL/AML) needs to be seen.^8^ Additionally, the incorporation of revumenib and other efficacious agents such as blinatumomab, as part of combination salvage regimens, to improve CR rates, increase MRD negative response rates and achieve better survival outcomes need to be studied in clinical trials. Trials that combine both these effective therapies are warranted. Finally, the use of NGS MRD monitoring platforms can further help to stratify patients with *KMT2A*-r B-ALL at different time points of their therapy and precisely identify those who might need a consolidative HSCT versus situations where it can be avoided.^51^ Such research is needed in R/R frontline settings as well.

## Allogeneic HSCT in *KMT2A*-r B-ALL

Allogeneic HSCT needs a mention in the context of *KMT2A*-r ALL. Large retrospective studies have not shown a survival benefit with HSCT in pediatric *KMT2A*-r B-ALL, especially t(4;11)-positive B-ALL.^5^ In a prospective Japanese study, early HSCT consolidation in infants with *KMT2A*-r ALL in first remission after intensive chemotherapy did not lead to particularly high OS (4-year EFS and OS rates of 43% and 62%, respectively).^52^ While HSCT may not be advocated in first remission to all pediatric patients with *KMT2A*-r B-ALL, it is often considered at this stage in infants and those with high-risk features and inadequate MRD clearance.^53^ In the MLL-10 trial of the Japanese Pediatric Leukemia/Lymphoma group only patients with age<180 days and/or CNS disease (high-risk group) were considered for allogenic HSCT. Among 56 such high-risk patients, 43 could be transplanted, leading to a 3-year EFS rate of 57% and OS of 80% in this group.^54^ The treatment regimen dictated a pharmacokinetically adjusted individualized dosing of busulfan along with cyclophosphamide and etoposide, which was the conditioning regimen administered to 38 of the 43 transplanted patients. Notably, 22% patients developed SOS, >90% of whom had proper busulfan levels.

The role of HSCT in CR1 in infants with *KMT2A*-r B-ALL needs to be revisited considering HSCT toxicities and long-term complications, especially given the availability of superior blinatumomab-containing regimens. Whether CAR T-cell therapy can be used as a consolidation or as an HSCT-sparing approach in high-risk *KMT2A*-r pediatric B-ALL, also needs to be studied. The ability to salvage these patients with novel agents such as menin inhibitors, blinatumomab and CAR T-cell therapy can also lead to HSCT being used in later lines of therapy. NGS MRD monitoring may enable a more precise selection of patients who can benefit the most from therapy intensification, including the frontline use of CAR T or allogeneic HSCT as well as post CAR T allogeneic HSCT.^44,55^

## Conclusions

The availability of novel agents with formidable efficacy in conventional high-risk B-ALL subtypes has been a promising improvement over SOC therapies which did not lead to favorable outcomes. *KMT2A*-r ALL is a prototype success story in the journey of improving outcomes of pediatric ALL. The recent data with blinatumomab, CAR T-cell therapy and menin inhibitors in infantile *KMT2A*-r B-ALL have shown astounding improvement in outcomes in this otherwise challenging disease subset. Combing these targeted approaches in frontline treatment to achieve deep responses and avoid HSCT in traditional high-risk subsets of *KMT2A*-r B-ALL needs to be studied in future clinical trials. Consideration towards long-term toxicity while curing the ALL also needs to remain in focus. The use of NGS MRD for disease monitoring can help identify patients who can benefit from treatment intensification or de-intensification.
